# Risk factors for lateral pelvic lymph node metastasis in patients with rectal neuroendocrine tumors: a systematic review and meta-analysis

**DOI:** 10.3389/fonc.2025.1500623

**Published:** 2025-01-31

**Authors:** Ziyue Chen, Dajian Zhu

**Affiliations:** Shunde Women and Children's Hospital of Guangdong Medical University, Foshan, Guangdong, China

**Keywords:** rectal neuroendocrine tumors, lateral pelvic lymph node metastasis, risk factors, treatment, meta-analysis

## Abstract

**Background and objective:**

Lateral pelvic lymph node (LPLN) metastasis is one of the prominent reasons for local recurrence in patients with rectal neuroendocrine tumors (RNETs). The evaluation criteria of lateral lymph node metastasis for patients and the indications and value of lateral pelvic lymph node dissection (LPLD) have been controversial. Total mesorectal excision (TME), a conventional surgical treatment for RNETs, excluding lateral lymph nodes, may be one of the reasons for postoperative local recurrence. This study aimed to analyze the risk factors for LPLN metastasis in patients with RNETs in order to guide surgical methods.

**Methods:**

We searched relevant databases (PubMed, Embase, Medline, Cochrane Library, and Web of Science) for articles published between 1 January 2000 and 1 April 2024 to evaluate the risk factors for LPLN metastasis in patients with RNETs in this meta-analysis.

**Results:**

A total of seven articles with 433 patients were included in this study. The overall results showed that a WHO grade > G1, tumor invasion of the muscularis propria or deeper, lymphovascular invasion (LVI), mesorectal lymph node metastasis (MLNM), and distant metastasis (M1) were significant risk factors for LPLN metastasis in patients with RNETs (P <0.05).

**Conclusion:**

This study identified key risk factors for LPLN metastasis in patients with RNETs, providing guidance for treatment strategies. A comprehensive evaluation of these risk factors and imaging findings is recommended to tailor personalized treatment strategies that optimize survival outcomes and improve quality of life.

**Systematic review registration:**

https://www.crd.york.ac.uk/prospero/, identifier CRD42024581891.

## 1 Introduction

Rectal neuroendocrine tumors (RNETs), which develop from rectal peptidergic neurons and neuroendocrine cells capable of producing amine hormones through intracellular processes, were previously known as “carcinoid tumors” and considered rare. However, over the past two decades, the incidence of neuroendocrine tumors in the gastrointestinal tract has risen significantly, with the highest increase observed in the rectum. This trend may be attributed to the growing use of gastrointestinal endoscopy and heightened awareness among healthcare professionals (1–3).

Previous studies have reported that 5.9%–10.9% of patients with RNETs develop lateral pelvic lymph node (LPLN) metastases after radical rectal resection, which is strongly associated with a poor prognosis (4, 5). Current guidelines for RNET treatment recommend total mesorectal excision (TME) for patients with mesorectal lymph node metastases (MLNMs); however, lateral lymph node dissection (LPLD) is not addressed, leaving its role controversial (6–8). No studies have specifically examined the risk factors for LPLN metastasis in patients with RNETs. Most research studies describe lymph node involvement as “regional lymph node metastasis” without distinguishing between MLNM and LPLN. Evidence suggests that RNET patients may exhibit skip metastasis, where LPLN metastases occur without MLNM involvement, indicating differing metastatic pathways (1, 9). Consequently, the risk factors for MLNM and LPLN metastasis should not be analyzed together.

To address this gap, we conducted a meta-analysis of all relevant studies to identify risk factors specific to LPLN metastasis. The findings aim to guide treatment strategies for RNET patients with suspected LPLN involvement.

## 2 Materials and methods

### 2.1 Literature search

**Figure 1:**
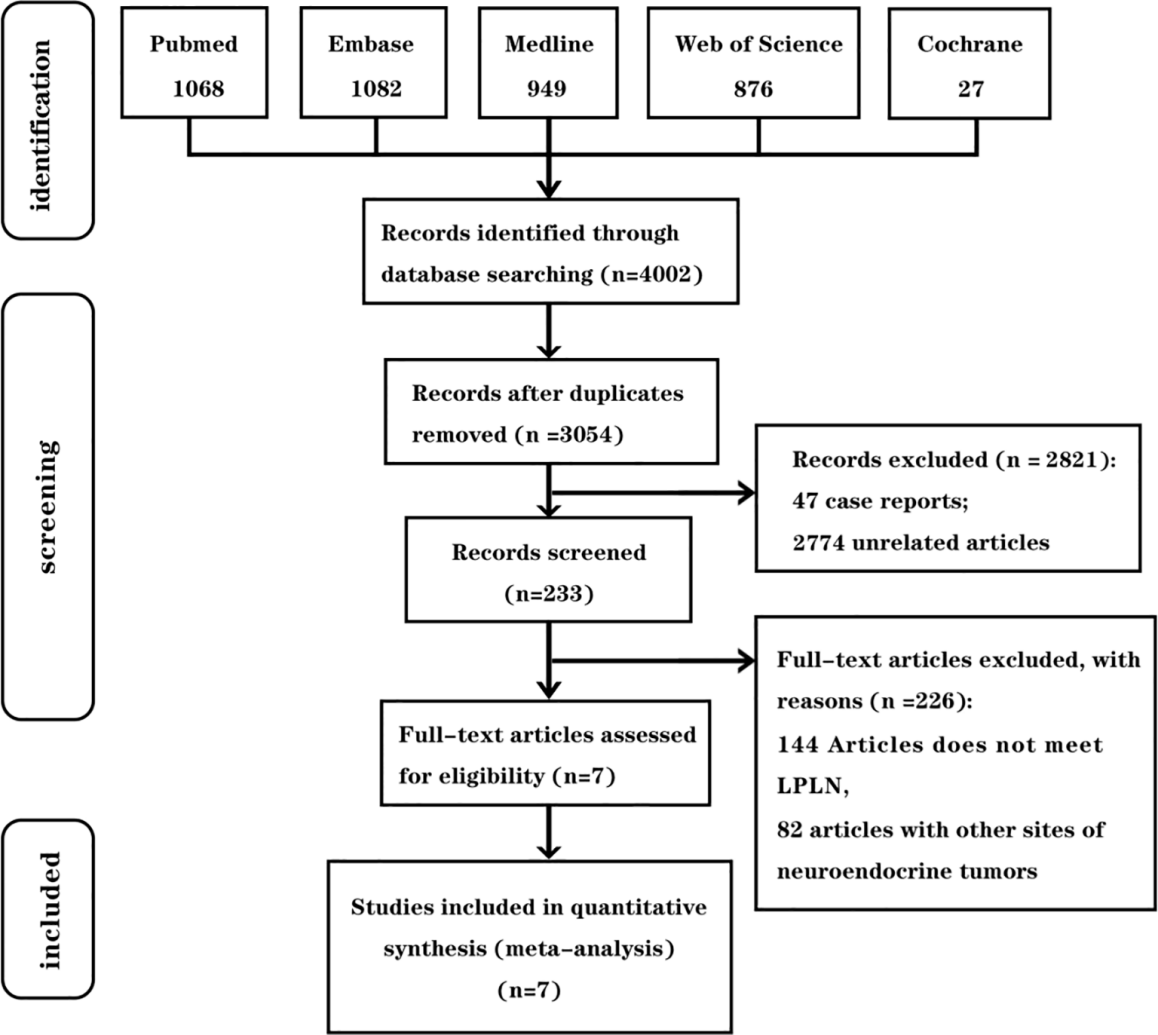
Inclusion and exclusion criteria chart.

We searched PubMed, Embase, Medline, Cochrane Library, and Web of Science for studies published between 1 January 2000 and 1 April 2024. The search strategy included Medical Subject Headings (MeSH) and free-text terms such as “rectal neuroendocrine tumor,” “lymph node metastasis,” and “risk factor.” No regional restrictions were applied and only human studies published in English were included. Relevant articles were identified through a careful review of the retrieved literature. The article screening process is outlined in **Figure 1**.

### 2.2 Article selection

The inclusion criteria were as follows:

1. Participants: Patients with an RNET and clinically suspected LPLN metastasis.
2. Intervention: Pathological examination confirmed positive LPLN metastasis.
3. Comparison: Pathological examination confirmed negative LPLN metastasis.
4. Outcome Measures: At least one of the following endpoints was reported: ① age, ② sex, ③ tumor size, ④ tumor grade (World Health Organization, WHO), ⑤ depth of tumor invasion, ⑥ lymphovascular invasion (LVI), ⑦ mesorectal lymph node metastasis (MLNM), ⑧ distant metastasis (M1).

Studies were excluded if they:

1. were duplicate publications;
2. were reviews, case reports, conference abstracts, or unrelated studies;
3. focused on lymph node metastasis unrelated to LPLN;
4. did not report any of the specified outcome measures.

### 2.3 Data extraction and endpoints

Two authors independently reviewed the included studies and extracted relevant data. The information collected included the study reference, first author, year of publication, country, number of patients, LPLN metastasis rate, and reported endpoints. Factors were considered as endpoints if data for them were available from at least two studies. The endpoints analyzed were ① age, ② sex, ③ tumor size, ④ tumor grade (WHO), ⑤ depth of tumor invasion, ⑥ LVI, ⑦ mesorectal lymph node metastasis, and ⑧ Distant metastasis (M1).

### 2.4 Study quality assessment

Two authors independently assessed the quality of the included studies using the Newcastle-Ottawa Scale (NOS), which assigns a maximum score of nine points per study (10). Studies scoring below six were classified as low-quality and excluded from the analysis. Any disagreements were resolved through discussion between the authors, and if consensus could not be reached, a third author was consulted to facilitate a resolution. This systematic review was conducted according to the Meta-analysis of Observational Studies in Epidemiology (MOOSE) guidelines and the PRISMA (Preferred Reporting Items for Systematic Reviews and Meta-Analyses) statement (11). It was registered with PROSPERO under the ID **CRD42024581891**.

### 2.5 Statistical analysis

Statistical analyses were conducted using RevMan 5.4 (Cochrane Collaboration) and Stata 18.0 software. To analyze dichotomous variables, odds ratios (ORs) with 95% confidence intervals (CIs) were used. Study heterogeneity was assessed using the Q-test p-value and the I² statistic. When the Q-test p-value was >0.1 and I² <50%, indicating low heterogeneity, a fixed-effects model was used to calculate the pooled estimate. Conversely, a random-effects model was applied when the Q-test p-value was <0.1 or I² >50%, suggesting significant heterogeneity. To ensure the robustness of the results, a leave-one-out sensitivity analysis was performed by sequentially excluding each study.

Although publication bias was initially assessed using funnel plots for visual inspection of symmetry, we have removed these from the results due to the limited number of included studies, which was insufficient to interpret the funnel plots reliably. Instead, statistical tests such as Egger’s or Begg’s test were applied for endpoints reported in five or more studies to evaluate publication bias further. Given the limited number of included studies for most endpoints (<10), the results of these tests should be interpreted with caution due to potentially low statistical power. The level of statistical significance was set at p <0.05, with heterogeneity tests considered significant at p <0.1.

## 3 Results

### 3.1 Study selection and flowchart

**Figure 1** outlines the article selection process. The database search initially yielded 4,002 articles, with no additional articles identified through reference review. After removing duplicates, 3,054 articles remained for screening by title and abstract. Of these, 2,821 were excluded for not meeting the inclusion criteria. A full-text review was conducted of 233 articles, ultimately leading to the inclusion of seven studies in this meta-analysis.

### 3.2 Basic features of the study

This study included seven retrospective articles comprising 433 patients, with LPLN positivity rates ranging from 2.56% to 27.27%. Most studies originated from Asia (two from Japan, one from Korea, and one from China), while two were conducted in Western countries (one in the UK and one in the USA). The key characteristics of these studies are summarized in **Table 1**.

**Table 1:** Main characteristics of the selected studies.

Author/Year of publication	Country	Case number	LPLN(+)rate	Outcome
Miyoshi et al., 2019 (4)	Japan	102	5.88% (6/102)	⑦⑧
Wang et al., 2016 (12)	America	22	27.27% (6/22)	①②③⑥⑦⑧
Kim et al., 2013 (13)	Korea	188	1.06% (2/188)	①②③④⑤⑥⑦⑧
Tsukamoto et al., 2008 (14)	Japan	23	13.04% (3/23)	①②③④⑤⑦⑧
Fujimoto et al., 2008 (15)	Japan	17	11.76% (2/17)	①②③⑤⑥⑦
Wang et al., 2022 (16)	China	42	14.29% (6/42)	④⑦
O’Neill et al., 2021 (17)	England	39	2.56% (1/39)	③④⑤⑥⑦⑧

① Age, ② Sex, ③ Tumor size, ④ Tumor grade ⑤ Depth of tumor invasion, ⑥ LVI, ⑦ MLNM ⑧ M1.

### 3.3 Quality assessment

**Table 2** shows that all the included articles achieved a score of six or higher, categorizing them as high-quality studies.

**Table 2:** The NOS scores of studies.

Included study	Selection	Comparability	Outcome	Score
Miyoshi et al., 2019 (4)	⭐⭐⭐	⭐⭐	⭐⭐⭐	8
Wang et al., 2016 (12)	⭐⭐⭐	⭐	⭐⭐⭐	7
Kim et al., 2013 (13)	⭐⭐	⭐	⭐⭐⭐	6
Tsukamoto et al., 2008 (14)	⭐⭐⭐	⭐	⭐⭐⭐	7
Fujimoto et al., 2008 (15)	⭐⭐⭐	⭐	⭐⭐⭐	7
Wang et al., 2022 (16)	⭐⭐⭐	⭐	⭐⭐⭐	7
O’Neill et al., 2021 (17)	⭐⭐⭐	⭐	⭐⭐⭐	7

The ⭐ represent the various scores of the NOS scale.

### 3.4 Outcomes

For all the outcomes, low statistical heterogeneity was observed among the studies, allowing the use of a fixed-effects model. The pooled results revealed a significantly higher risk of LPLN metastasis associated with a WHO grade > G1 (OR = 7.76; 95% CI: 2.01–29.94; *I²* = 44%), tumor invasion reaching the muscularis propria or deeper (OR = 4.51; 95% CI: 1.29–15.75; *I²* = 36%), LVI (OR = 4.97; 95% CI: 1.22–20.22; *I²* = 0%), MLNM (OR = 4.07; 95% CI: 1.73–9.53; *I²* = 13%), and M1 (OR = 4.07; 95% CI: 1.73–9.53; *I²* = 0%). These factors were identified as significant risk factors for LPLN metastasis.

#### 3.4.1 Age

**Figure 2:**
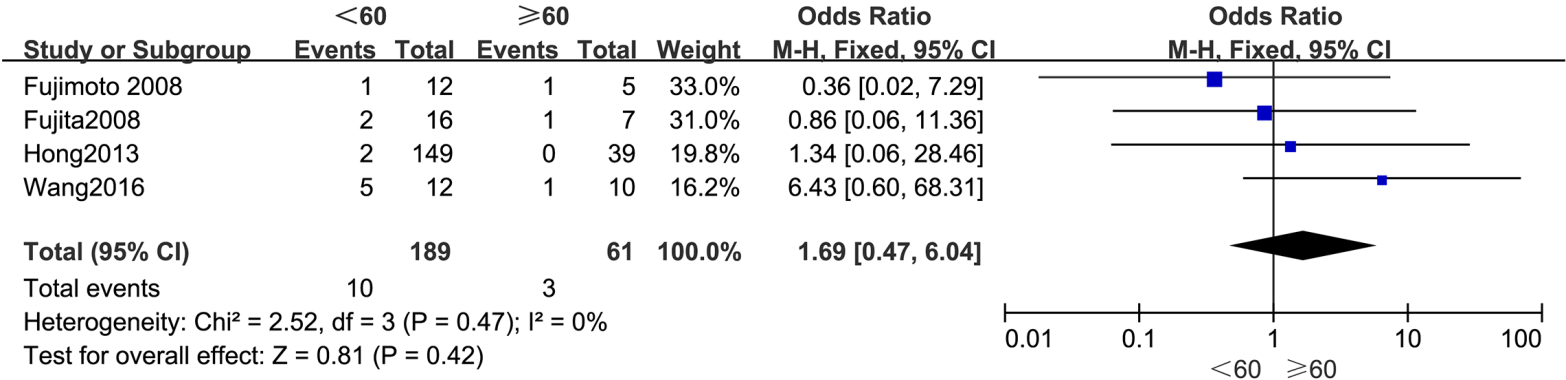
Forest plot of age.

**Figure 2** summarizes the outcomes. Due to low statistical heterogeneity (*I²* = 0%, *p* > 0.1), a fixed-effects model was applied. The pooled analysis indicated that age did not significantly impact LPLN metastasis (OR = 1.69; 95% CI: 0.47–6.04; *p* > 0.05).

#### 3.4.2 Sex

**Figure 3:**
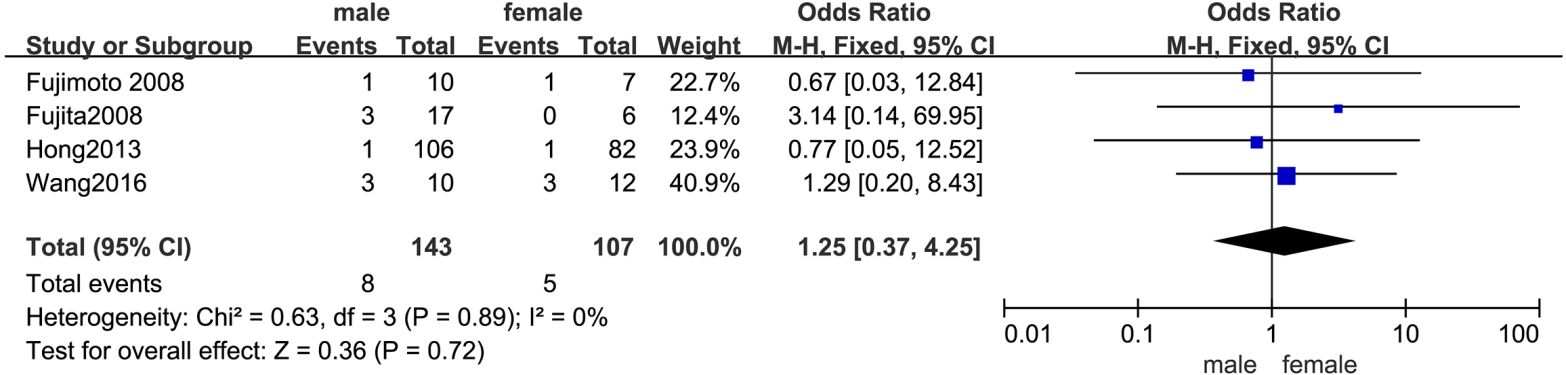
Forest plot of sex.

The outcomes are presented in **Figure 3**. Given the low statistical heterogeneity (*I²* = 0%, p > 0.1), a fixed-effects model was utilized. The pooled analysis indicated that sex did not significantly influence the likelihood of LPLN metastasis (OR = 1.25; 95% CI: 0.37–4.25; *p* > 0.05).

#### 3.4.3 Tumor size

**Figure 4:**
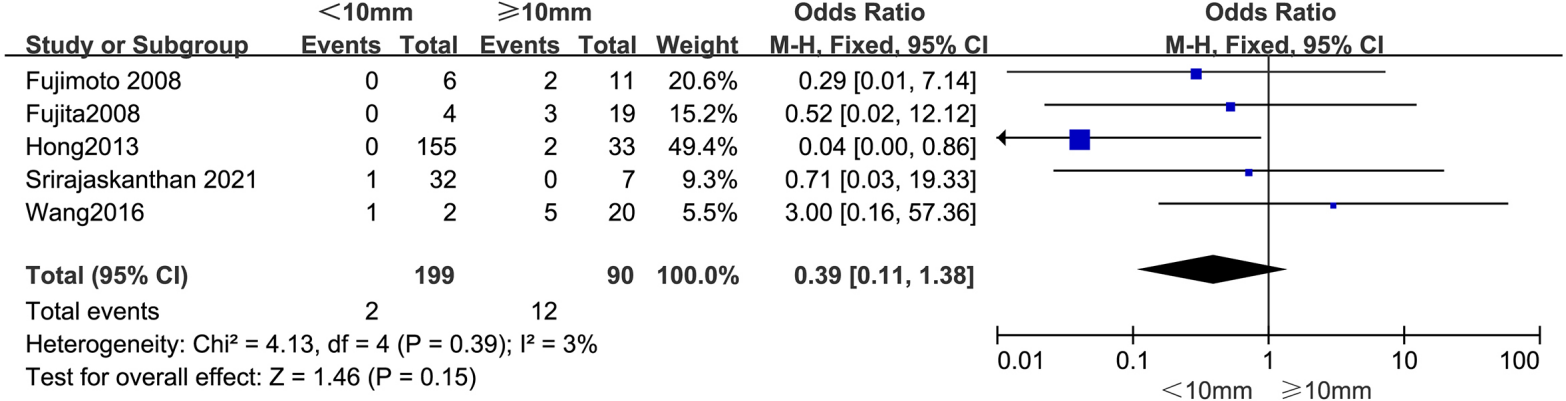
Forest plot of tumor size.

The outcomes are summarized in **Figure 4**. Due to low statistical heterogeneity (*I²* = 3%, *p* > 0.1), a fixed-effects model was applied. The pooled analysis indicated that tumor size did not significantly impact the likelihood of LPLN metastasis (OR = 0.39; 95% CI: 0.11–1.38; *p* > 0.05).

#### 3.4.4 Tumor grade (WHO)

**Figure 5:**
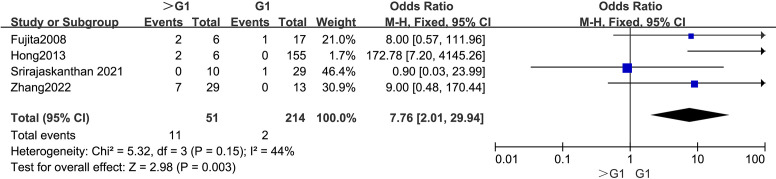
Forest plot of tumor grade.

The outcomes are summarized in **Figure 5**. Due to low statistical heterogeneity (*I²* = 44%, *p* > 0.1), a fixed-effects model was applied. The analysis demonstrated a strong association between a WHO grade > G1 and LPLN metastasis (OR = 7.76; 95% CI: 2.01–29.94; *p* < 0.05).

#### 3.4.5 Depth of tumor invasion

**Figure 6:**
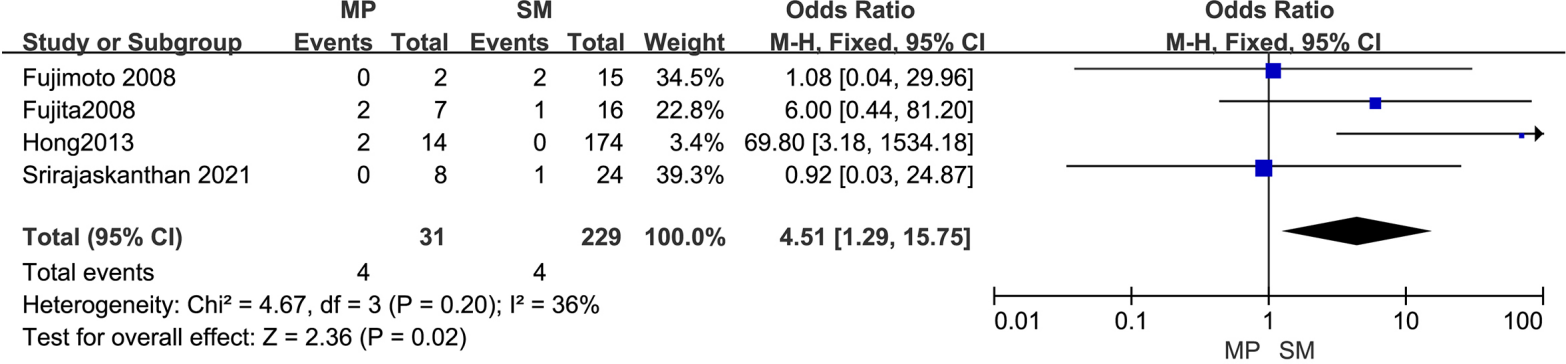
Forest plot of depth of tumor invasion.

The outcomes are summarized in **Figure 6**. Due to low statistical heterogeneity (*I²* = 36%, *p* > 0.1), a fixed-effects model was applied. The analysis showed a strong association between tumor invasion into the muscularis propria or deeper and LPLN metastasis (OR = 4.51; 95% CI: 1.29–15.75; *p* < 0.05).

#### 3.4.6 LVI

**Figure 7:**
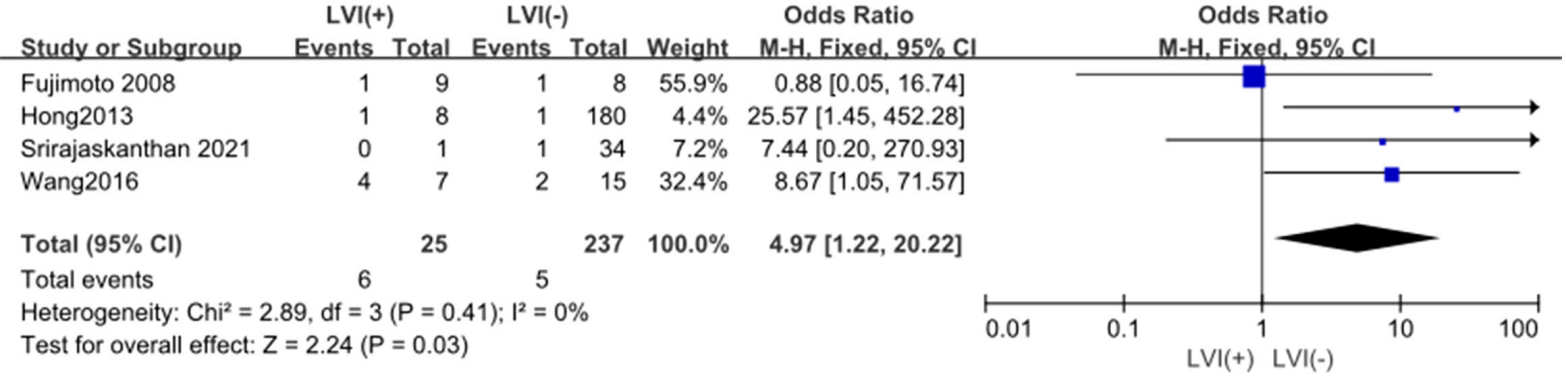
Forest plot of LVI.

The outcomes are summarized in **Figure 7**. A fixed-effects model was applied due to low statistical heterogeneity (*I²* = 0%, *p* > 0.1). The analysis demonstrated a strong association between LVI and LPLN metastasis (OR = 4.97; 95% CI: 1.22–20.22; *p* < 0.05).

#### 3.4.7 MLNM

**Figure 8:**
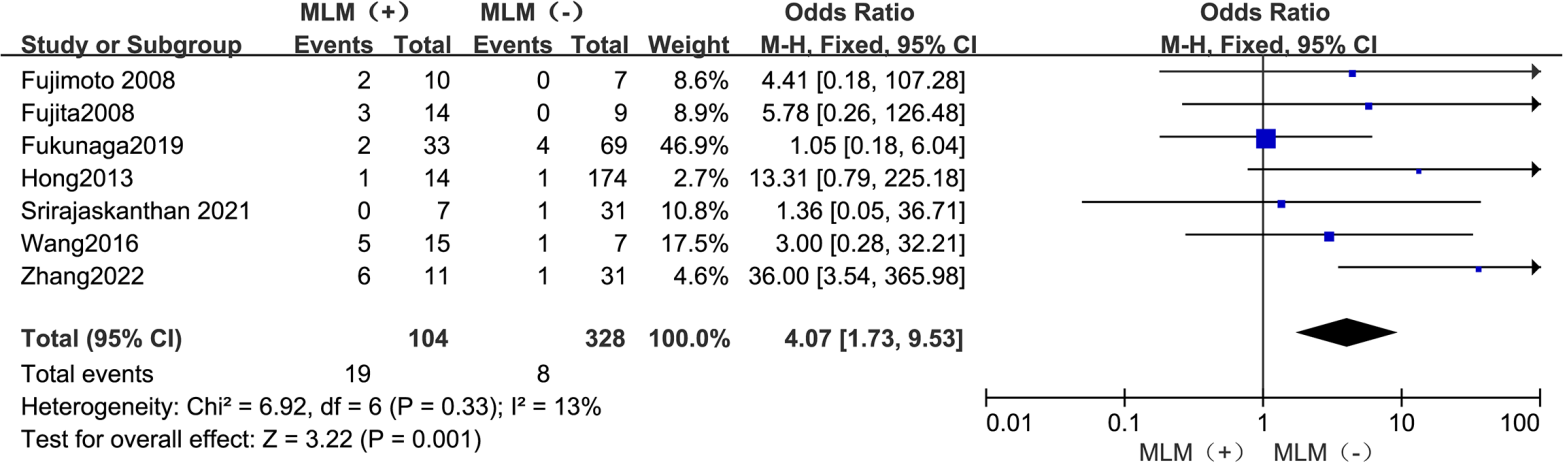
Forest plot of MLNM.

The outcomes are summarized in **Figure 8**. Due to low statistical heterogeneity (*I²* = 13%, *p* > 0.1), a fixed-effects model was used. The analysis showed a significant association between MLNM and LPLN metastasis (OR = 4.07; 95% CI: 1.73–9.53; *p* < 0.05).

#### 3.4.8 M1

**Figure 9:**
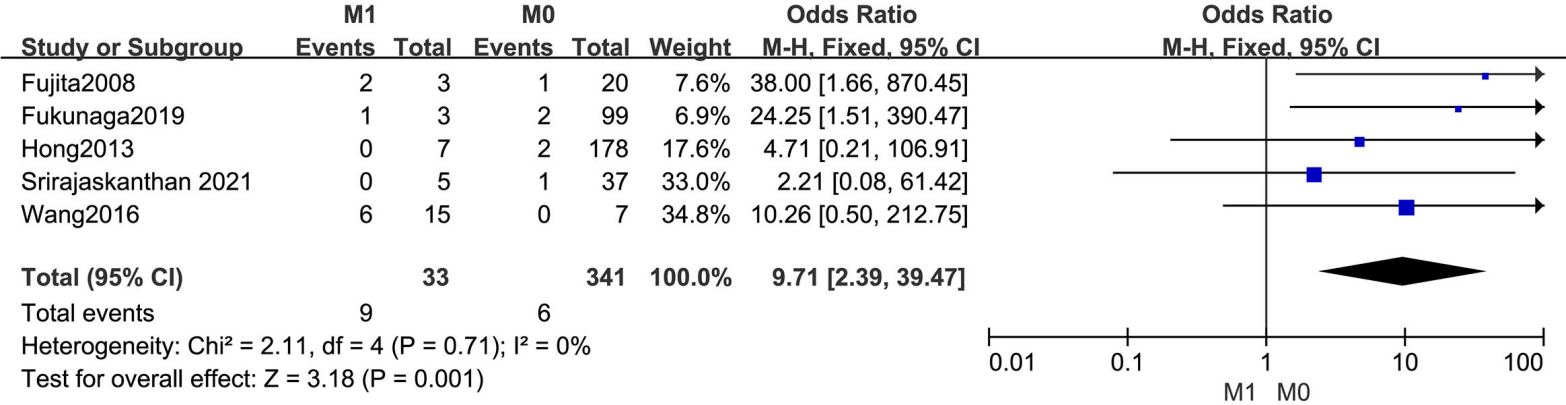
Forest plot of M1.

The outcomes are summarized in **Figure 9**. Due to low statistical heterogeneity (*I²* = 0%, *p* > 0.1), a fixed-effects model was employed. The analysis revealed a strong association between M1 and LPLN metastasis (OR = 9.71; 95% CI: 2.39–39.47; *p* < 0.05).

### 3.5 Sensitivity analysis

**Figure 10:**
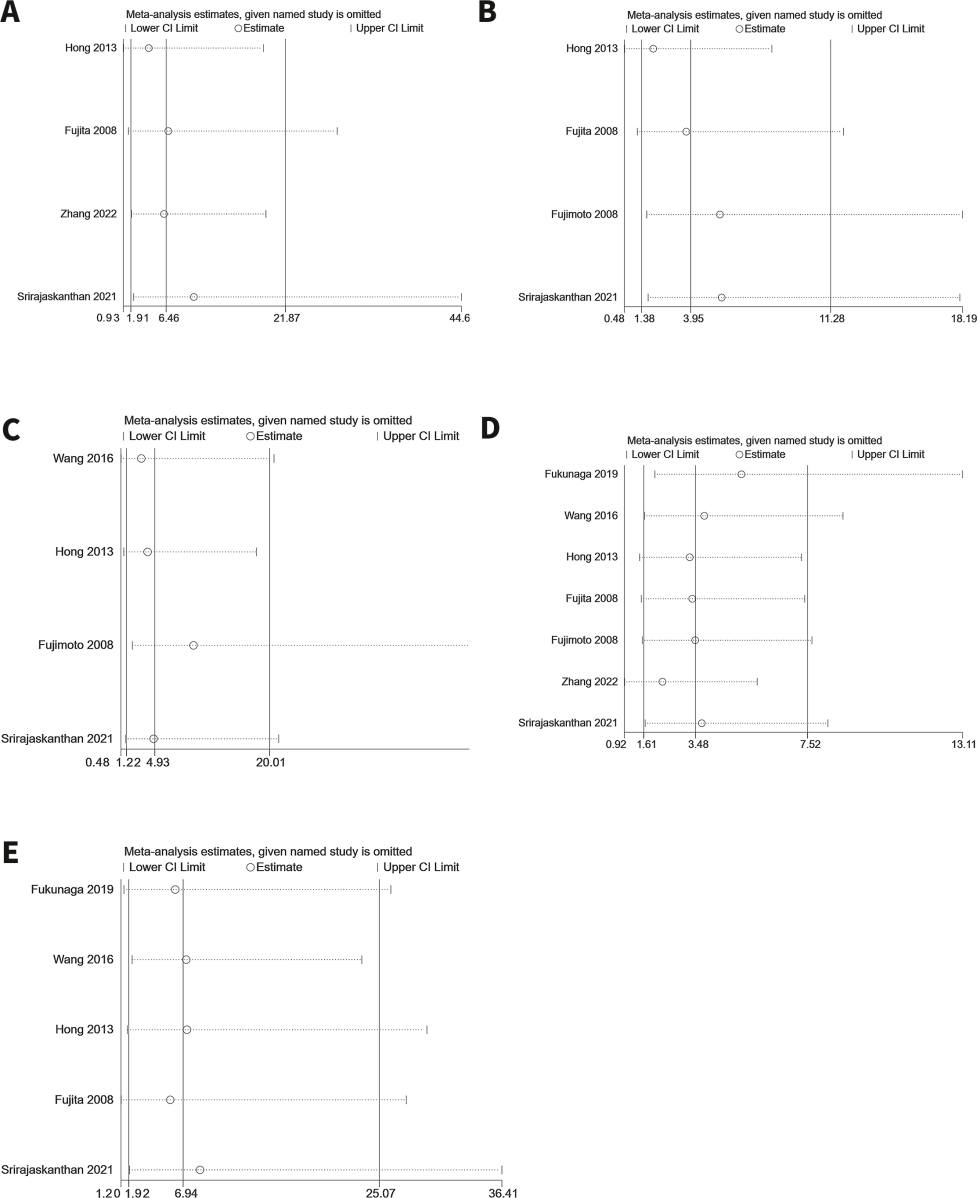
Sensitivity analysis. **(A)** Tumor grade **(B)** Depth of tumor invasion **(C)** LVI **(D)** MLNM **(E)** M1.

Sensitivity analyses were conducted for significantly influential endpoints to evaluate the robustness of the results. A leave-one-out sensitivity analysis was performed to assess the impact of each study on the pooled outcomes. The analysis confirmed that none of the results were significantly affected by any single study, indicating the overall reliability of this meta-analysis. The results of the sensitivity analysis are displayed in **Figure 10**.

### 3.6 Publication bias

To ensure the validity of the meta-analysis results, we assessed publication bias for significant impact factors using Egger’s and Begg’s tests for endpoints with at least five studies. No significant publication bias was detected for articles addressing MLNM (Egger’s test: *p* = 0.3846; Begg’s test: *p* = 0.7639). Similarly, no significant publication bias was observed for articles addressing M1 (Egger’s test: *p* = 0.3484; Begg’s test: *p* = 0.4624). For other endpoints, publication bias was evaluated through funnel plots. Overall, the results indicated no significant publication bias for any of the analyzed factors, supporting the reliability of the findings. Regression analysis was not conducted, as fewer than 10 studies were included.

## 4 Discussion

Advances in surgical techniques and multidisciplinary treatments in recent years have significantly improved the prognosis of patients with RNETs while reducing local recurrence rates. In rectal cancer, the evidence indicates that the most common site of local recurrence is LPLN metastasis (18). Based on this, we hypothesized that LPLN metastasis may also be the primary cause of local recurrence in RNET, particularly with the widespread adoption of TME and multidisciplinary approaches. Studies have shown that LPLN metastasis in RNET is directly associated with poor prognosis (19). Despite this, the current guidelines for diagnosing and treating RNETs lack specific strategies for addressing LPLN metastasis. Given the significant influence of lymph node metastasis and its risk factors on treatment decisions, there is a clear need to establish practical diagnostic and therapeutic approaches for LPLN involvement in RNET. To address this gap, we investigated the risk factors for LPLN metastasis in RNET to provide evidence-based guidance for clinical management.

### 4.1 Etiology of LPLN metastasis

LPLN metastasis may arise due to several factors: (1) obstruction of the rectal mesenteric lymphatic pathway by a large primary tumor, leading to the development of alternative lymphatic pathways; (2) alterations in lymphatic drainage patterns caused by tumor invasion of the lymphatic system; and (3) prior surgical procedures, which may contribute to metastasis through direct tumor cell implantation or scar tissue formation in the surgical bed within the mesorectal envelope, potentially resulting in the establishment of an alternative extramesorectal lymphatic drainage route (12, 20).

### 4.2 Risk factors for LPLN metastasis

This study utilized a meta-analysis to identify risk factors for LPLN metastasis, aiming to provide a scientific basis for assessing the need for lateral lymph node dissection in RNET. The analysis revealed that tumor grade (WHO > G1), tumor invasion into the muscularis propria or deeper, LVI, MLNM, and M1 were significant risk factors for LPLN metastasis. Conversely, age, sex, and tumor size were not found to be predictive of LPLN involvement.

#### 4.2.1 Tumor size and lymphovascular invasion

Previous studies have often identified tumor size ≥10 mm as a key parameter for lymph node metastasis in RNET (21–23). Using a 10 mm cut-off in our study, we found that tumor size was not a significant risk factor for LPLN metastasis. This discrepancy may reflect the highly heterogeneous biological behavior of RNETs, which are generally slow-growing, low-invasive, and frequently smaller than 10 mm. However, lymph node metastasis can occur even in tumors smaller than 10 mm (24). Unlike rectal cancer, RNETs originate from Kultschitzky cells in the deep mucosa, an area rich in lymphatic vessels, potentially leading to early LVI and smaller metastatic lymph nodes (4, 25). Histological evidence of tumor cells in lymphatic or blood vessels indicates a highly invasive nature, increasing the risk of LPLN metastasis. These findings emphasize the need to monitor LPLN metastasis even in small RNETs, particularly in those with LVI.

#### 4.2.2 Tumor grade

Tumor grade, reflecting cell differentiation and malignancy potential, predicts aggressive behavior in RNETs. Studies consistently identify a tumor grade > G1 as a risk factor for metastasis (26, 27). In our analysis, we confirmed that a tumor grade > G1 is associated with a higher risk of LPLN metastasis. Therefore, patients with RNETs of grades > G1 should be carefully evaluated for potential LPLN involvement.

#### 4.2.3 Tumor invasion depth

Depth of invasion, often assessed using endorectal ultrasound, significantly influences the likelihood of lymphatic spread. Tumors invading the muscularis propria are more prone to lymphatic dissemination, increasing the risk of LPLN metastasis. Consistent with earlier research (28–30), our findings indicate that deeper invasion, particularly to the muscularis propria, is a significant risk factor for LPLN metastasis in patients with RNETs.

#### 4.2.4 MLNM and M1

Our study found that MLNM is a significant risk factor for LPLN metastasis despite prior studies suggesting the possibility of “skip metastasis” in RNETs, where LPLN metastasis occurs without MLNM involvement (4, 9). This may be due to tumor invasion of lymphatic vessels or the development of alternative lymphatic pathways following the obstruction of the mesenteric lymphatic ducts by enlarged lymph nodes. Similarly, M1 was identified as a decisive risk factor for LPLN metastasis, underscoring the importance of vigilant monitoring in RNET patients with distant disease.

### 4.3 Indication of LPLN metastasis

Imaging is central in the preoperative diagnosis of LPLN metastasis in RNET. However, due to the low malignant potential and slow growth of RNETs, metastatic LPLNs are often small, making detection by computed tomography (CT) or magnetic resonance imaging (MRI) challenging (4). The Japan Cancer Institute previously recommended considering LPLD when preoperative CT or MRI identifies lymph nodes with a longitudinal diameter greater than 7 mm. In a retrospective study of 102 patients with RNETs who underwent radical surgery, six out of seven patients who underwent LPLD were found to have lymph node metastasis, yielding a positive rate of 86% under this criterion (4).

Additionally, when preoperative imaging results for lymph node metastasis are inconclusive or potentially inaccurate, advanced imaging techniques such as 68Gallium positron emission tomography (PET-CT) or somatostatin-receptor scintigraphy (SRS) can improve diagnostic accuracy. These modalities can help confirm the presence of metastatic lymph nodes and guide surgical planning (31, 32).

Beyond imaging, molecular and genetic biomarkers are emerging as valuable tools for assessing the risk of LPLN metastasis. Research has highlighted the potential of microRNAs (miRNAs) such as miR-21 and miR-222, which are associated with tumor invasion and metastasis. Specifically, miR-21 has been linked to lymph node metastasis in gastrointestinal neuroendocrine tumors, making it a promising candidate for evaluating LPLN involvement (33). Additionally, the combined expression of miR-186 and its downstream target PTTG1 (pituitary tumor-transforming 1) has shown predictive value for tumor infiltration and invasion in colorectal neuroendocrine tumors, with detection possible through non-invasive blood and stool tests (34).

Other molecular markers, including CpG island methylator phenotype (CIMP) and miR-885-5p, have been associated with lymphovascular invasion in rectal neuroendocrine tumors, a critical risk factor for LPLN metastasis (35). These findings suggest that molecular profiling could identify high-risk patients who might benefit from LPLD, even when imaging results are borderline or inconclusive.

Integrating molecular biomarkers into preoperative evaluations could support a more personalized approach to patient care by complementing imaging findings. Further research is needed to validate these biomarkers in larger, multicenter studies and to incorporate them into existing diagnostic workflows effectively.

### 4.4 Treatment modalities

Surgery remains the primary treatment for RNETs. According to the 2023 European Neuroendocrine Tumor Society (ENETS) guidelines for colorectal neuroendocrine tumors, radical surgery is recommended for patients with RNETs under the following conditions: (1) imaging suggests lymph node metastasis, (2) tumor size > 2 cm, or (3) tumor size of 1–2 cm with R1 resection after endoscopic resection, or tumors classified as high grade or G3 (6). Radical surgery involves complete tumor excision and regional lymph node dissection, which includes TME and, in some cases, LPLD.

While TME is a widely accepted standard for RNET treatment and is explicitly addressed in the current guidelines (6–8), LPLD remains a subject of debate, with no clear consensus or guideline for its use in RNET patients. The treatment of RNET patients with LPLN metastasis is primarily guided by the therapeutic strategies used for LPLN metastasis in rectal cancer. However, the treatment of LPLN metastasis of rectal cancer is also controversial. Western scholars argue that LPLD is associated with complications such as prolonged surgery, increased intraoperative blood loss, and postoperative effects on urinary and sexual function without evidence of improved survival. Consequently, neoadjuvant chemoradiotherapy (NCRT) and TME are the preferred standard treatments in Western countries (36).

In contrast, Japanese studies classify LPLNs as regional lymph nodes in rectal cancer. Research demonstrates that LPLD significantly reduces local recurrence rates and improves 5-year survival outcomes. As a result, LPLD is a standard procedure in Japan (37). Similarly, the latest Chinese guidelines for rectal cancer recommend a comprehensive approach combining NCRT, TME, and selective LPLD for clinically diagnosed LPLN metastasis (38).

However, RNET and rectal cancer are distinct diseases, and metastatic lymph nodes in RNET are significantly smaller than those in rectal adenocarcinoma (4). Therefore, applying rectal cancer treatment strategies to LPLN metastasis in RNET may not be appropriate. Additionally, no evidence supports the use of NCRT for RNET patients with LPLN metastasis. Reports indicate that RNET patients with suspected LPLN metastasis who underwent LPLD had favorable outcomes, with no evidence of local recurrence during follow-ups ranging from 10 to 288 months (4, 15, 32, 39–47).

**Figure 11:**
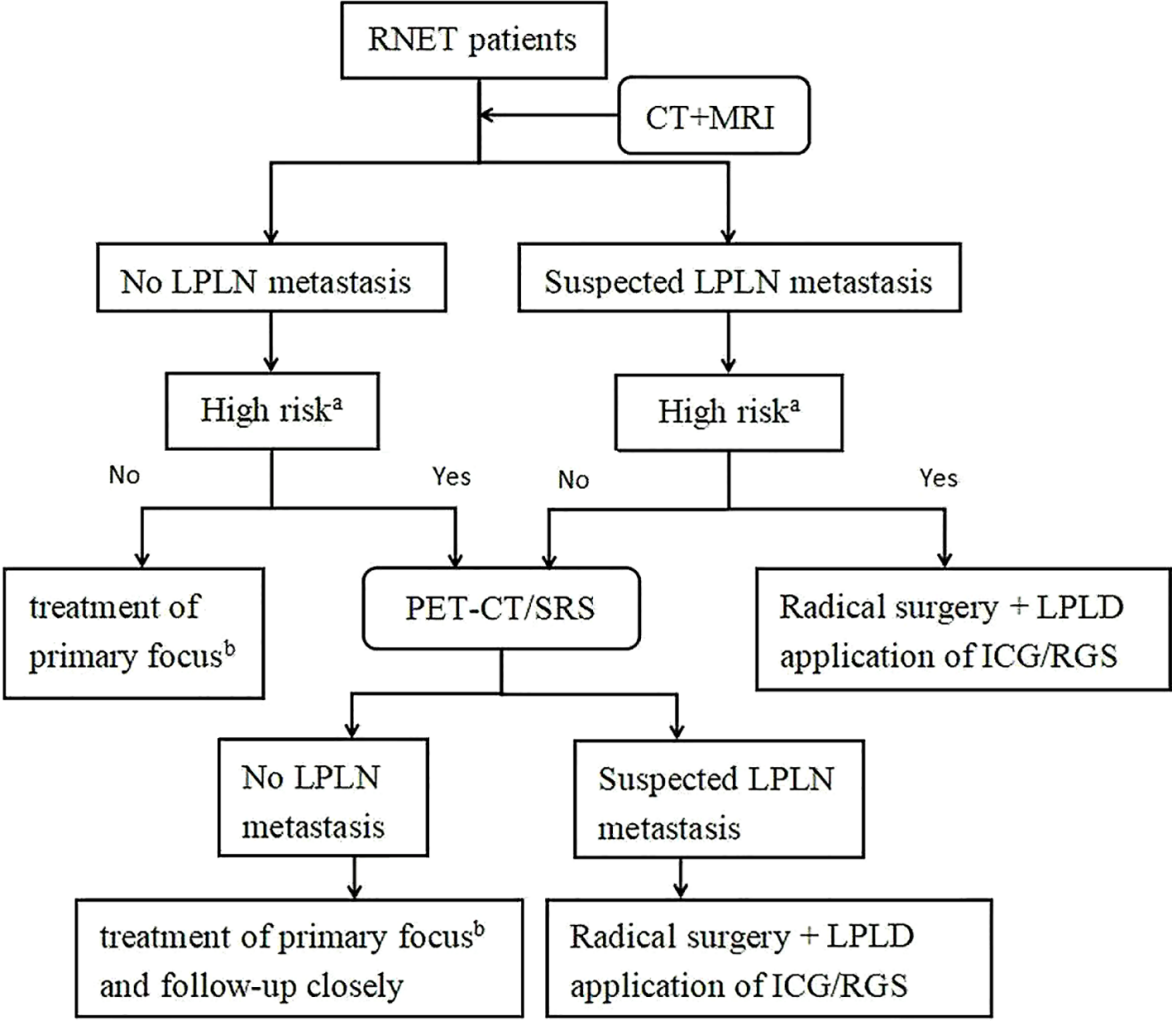
Treatment strategies for LPLN metastasis in patients with RNET. a. High risk refers to the factors noted in this study that influence LPLN metastasis including: tumor grade (WHO) > G1, tumor invasion of the muscularis propria or deeper, LVI, MLNM, and M1. b. Treatment of primary focus according to ENETS2023 guidelines, including endoscopic resection, transanal resection, and radical surgery (6). ICG, Indocyanine Green Fluorescence-Guided Imaging; RGS, Radioguided Surgery; RNET, Rectal Neuroendocrine Tumor; CT+MRI, Computed Tomography and Magnetic Resonance Imaging; LPLN, Lateral Pelvic Lymph Node; PET-CT/SRS, Positron Emission Tomography-Computed Tomography/Somatostatin Receptor Scintigraphy; LPLD, Lateral Pelvic Lymph Node Dissection.

Based on current evidence, we recommend incorporating LPLD for RNET patients with suspected LPLN metastasis and high-risk features identified through imaging and clinical assessment, as outlined in **Figure 11**.

### 4.5 Preoperative methods for localizing metastatic LPLNs

Several preoperative techniques for detecting LPLN metastasis in RNET have been explored, including radio-guided surgery (RGS) and indocyanine green (ICG) fluorescence-guided imaging. In a study by Wang et al., 14 patients received injections of 1 mCi technetium (99mTc) or indium (111In) pentetreotide into 3 to 4 sites around the tumor via preoperative colonoscopy. Of these, six patients were identified with positive LPLN metastasis and underwent LPLD. Postoperative pathology confirmed LPLN metastasis and the metastatic nodes were safely resected under RGS. Follow-up revealed no increased risk of sexual or urinary dysfunction in the patients who underwent LPLD (12). Similarly, Zhang et al. reported using ICG fluorescence-guided imaging in a patient with a RNET and suspected LPLN metastasis. Despite negative findings on preoperative CT, EUS, and PET-CT, 1 mL (2.5 mg) of ICG was injected into four locations (above, below, left, and right of the tumor) under an anoscope before surgery. Intraoperatively, fluorescence highlighted the left LPLN, which was resected during LPLD. Pathology confirmed metastasis, and the patient experienced no postoperative sexual or urinary dysfunction (5).

Both methods improved the detection of metastatic lymph nodes and effectively guided surgical resection in cases where imaging failed to identify lymph node involvement. Furthermore, no significant postoperative complications were reported following LPLD in these studies. Beyond these case studies, evidence from larger studies in rectal cancer patients undergoing LPLD supports the utility of ICG fluorescence-guided imaging. Retrospective studies with propensity-matched cohorts have shown that ICG significantly increases the number of harvested LPLNs, reduces intraoperative blood loss, and shortens operative times compared with LPLD alone (48, 49). Additionally, meta-analyses of ICG-guided imaging during LPLD highlight its safety and feasibility, with findings suggesting a reduction in postoperative complications, such as urinary retention, and an overall improvement in local control of the lateral pelvis (50).

While these findings are encouraging, most studies on ICG and RGS have been conducted in rectal cancer or are limited to a small case series in RNET. Larger, multicenter studies with diverse patient populations are needed to validate these results and determine their generalizability to patients with RNETs. Future research should also explore whether these techniques can reliably improve long-term oncological outcomes, such as overall survival and recurrence rates.

### 4.6 Limitations

Our study has several limitations. First, most of the included articles were retrospective, which may introduce selection and information biases. These studies often rely on inconsistent or incomplete patient records, potentially affecting the reliability of our findings. Second, the follow-up durations of the cases varied across studies, making it difficult to assess the long-term effectiveness of treatments such as LPLD. Furthermore, limited data on treatment outcomes prevented us from conducting a thorough risk-benefit analysis of LPLD, an area that warrants further investigation. Third, while we suggested PET-CT as a preferred imaging method for detecting LPLN metastasis (**Figure 11**), this recommendation was based on clinical observations and prior reports rather than direct comparisons with enhanced CT or MRI. The lack of head-to-head data on diagnostic performance weakens the strength of this conclusion and should be interpreted with caution. Fourth, although we identified several potential risk factors for LPLN metastasis, including WHO grade > G1, LVI, and MLNM, the small sample size limited our ability to conduct multivariate analysis. As a result, we could not determine which factors are independent predictors, reducing the clarity of their relative significance. Fifth, we were unable to compare LPLD with other treatment strategies, such as observation or alternative dissection approaches, due to a lack of available data. This absence of comparative analysis limits our ability to definitively assess the superiority of LPLD. Finally, most of the studies included in our analysis originated from Eastern countries, with only two from Western regions. This geographical imbalance may bias our findings toward regional clinical practices, limiting their generalizability to broader populations.

### 4.7 Future research prospects

Future research should focus on identifying risk factors for LPLN metastasis in RNET through advanced imaging, tumor markers, and genetic analysis. These efforts will help build more accurate prediction models, enabling earlier detection and better identification of high-risk patients. Large, multi-center, and long-term studies with diverse patient cohorts are needed to refine the indications for LPLD. Such studies should also assess the effectiveness of LPLD compared to other strategies, such as observation or neoadjuvant therapies, to guide clinical decision-making and improve treatment outcomes. Advancements in surgical techniques offer promising opportunities to enhance the precision and safety of LPLN dissection. Real-time navigation, ICG fluorescence imaging, and robotic-assisted surgery (RAS) could minimize complications, improve lymph node detection, and ultimately enhance survival rates and post-surgical quality of life. Further research should also investigate the role of neoadjuvant radiotherapy, targeted therapies, and immunotherapy as potential adjuncts to surgery. Exploring these approaches may reduce tumor burden, improve local control, and lessen the need for extensive surgical intervention. Finally, international collaboration is essential to address geographical biases, expand data sharing, and ensure findings are applicable across diverse populations. By combining innovative technologies, emerging therapies, and robust clinical research, future efforts can transform the management of LPLN metastasis in RNET, ultimately improving patient care and outcomes.

## 5 Conclusion

This study analyzed seven articles involving 433 patients to identify risk factors for LPLN metastasis in RNET. The meta-analysis revealed that tumor grade (WHO > G1), invasion into the muscularis propria or deeper, LVI, MLNM, and M1 were significant risk factors for LPLN metastasis. While LPLD can reduce local recurrence rates, it carries a risk of surgery-related complications.

## Data Availability

The datasets presented in this study can be found in online repositories. The names of the repository/repositories and accession number(s) can be found in the article/supplementary material.
